# Open Avulsion of the Achilles Tendon Through the Popliteal Fossa Without Associated Fracture: A Report of a Unique Case

**DOI:** 10.7759/cureus.111705

**Published:** 2026-06-29

**Authors:** Diogo Rodrigues, Ana Lucinda Correia, Filipa Adan e Silva, Cláudia Rodrigues, Pedro Leite

**Affiliations:** 1 Orthopaedics and Traumatology, Unidade Local de Saúde de Santo António, Porto, PRT

**Keywords:** achilles tendon avulsion, high-energy soft-tissue trauma, open tendon injury, orthopaedic trauma case report, popliteal fossa, suture anchor reinsertion

## Abstract

We present the case of a 58-year-old woman who sustained an open avulsion of the Achilles tendon with an unusual posterior extrusion through the popliteal fossa following a run-over trauma, remarkably without associated bony injury or major arterial damage. Surgical exploration revealed a complete tendon avulsion accompanied by a small venous laceration, which was successfully repaired. The tendon was anatomically reinserted into the calcaneus using suture anchors after subcutaneous tunneling along the posterior leg. The popliteal and distal wounds were closed primarily; the posterior wound through which the tendon had extruded subsequently developed necrosis and required delayed skin grafting for definitive coverage, which healed uneventfully. At two years, the wounds were fully healed, and radiographs confirmed a maintained reinsertion without degenerative change, although residual plantarflexion weakness persisted. Functional outcomes were documented with validated and objective measures: Achilles Tendon Total Rupture Score (ATRS) 72/100 and American Orthopaedic Foot & Ankle Society (AOFAS) Ankle-Hindfoot score 77/100 (fair), with six single-leg heel raises on the injured side versus 16 contralaterally. The absence of pre-injury baseline outcome scores is acknowledged as a limitation. To our knowledge, this injury pattern has not previously been reported, and the case underscores the importance of early, multidisciplinary management in rare and complex soft-tissue trauma.

## Introduction

Acute Achilles tendon rupture is among the most common tendon injuries of the lower limb, and population-based studies report a rising incidence over recent decades. Nationwide registry data from Scandinavia describe incidences of approximately 27 to 35 per 100,000 person-years [1,2], whereas a registry study based on United States emergency-department coding reports substantially lower figures [3]. This variability largely reflects differences in case ascertainment, healthcare setting, and study population rather than a true 20-fold difference in disease frequency. The overwhelming majority are closed injuries occurring at the relatively hypovascular mid-substance, approximately two to six centimetres proximal to the calcaneal insertion, and less commonly at the insertion itself or the proximal musculotendinous junction [3].

Rarer variants have only recently been characterised, including avulsion of the tendon from the gastrocnemius-soleus origin [4] and insertional sleeve avulsions, the latter typically associated with pre-existing insertional tendinopathy and Haglund’s deformity [5]. Open Achilles tendon ruptures are considerably less frequent and usually result from sharp lacerations, penetrating trauma, or high-energy mechanisms such as road-traffic accidents; even within this group, injuries cluster around the distal tendon and heel and carry substantial soft-tissue compromise and an elevated risk of infection [6,7].

Anatomically, the Achilles tendon lies within the superficial posterior compartment of the leg, tethered distally to the calcaneus and proximally continuous with the gastrocnemius-soleus muscle bellies, which are bounded by the deep crural fascia. The popliteal fossa lies proximal and deep to this compartment, separated from it by fascial planes and traversed by the neurovascular bundle. Proximal migration of a ruptured Achilles tendon into the popliteal fossa is therefore biomechanically unexpected, as it requires failure at or near the musculotendinous origin together with disruption of the intervening fascial boundaries. To our knowledge, an open Achilles tendon avulsion that retracts and extrudes proximally through the popliteal fossa, in the absence of any accompanying fracture or dislocation, has not previously been described. The rarity of this presentation posed distinct diagnostic and reconstructive challenges and is the focus of the present report.

## Case presentation

**Figure 1:**
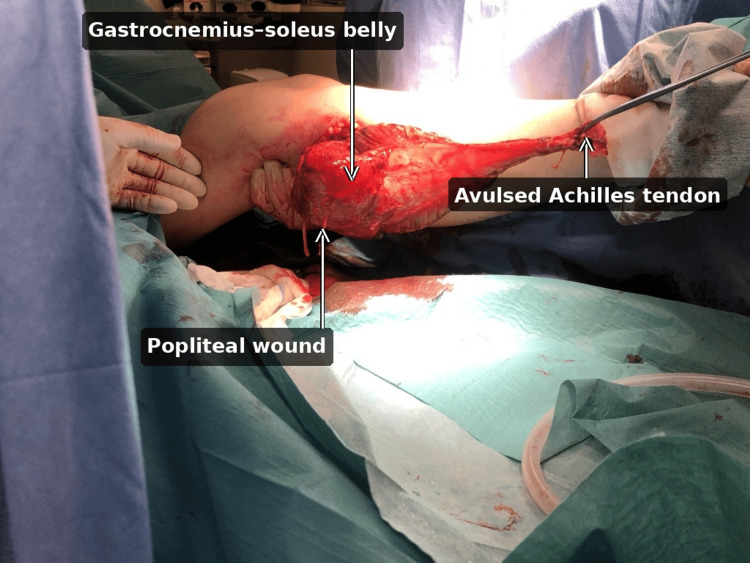
Open injury of the posterior compartment of the leg. The avulsed Achilles tendon (held by the forceps) is seen after extruding proximally through the popliteal wound; the gastrocnemius–soleus muscle belly and the popliteal wound are labelled for orientation.

A 58-year-old woman was admitted to the emergency department after being run over by a bus. On arrival, she was alert, haemodynamically stable, and showed no evidence of head, thoracic, or abdominal injury. Her left leg presented with a large open wound in the popliteal region, with active bleeding and exposure of deep muscular structures; the Achilles tendon was visibly protruding through the wound (Figure 1). There was extensive soft-tissue degloving of the posterior compartment overlying the gastrocnemius muscle bellies, together with a degloving injury of the anteromedial foot. Distal neurovascular examination was normal, with intact pedal pulses, normal capillary refill, and no sensorimotor deficit in the tibial or peroneal nerve distributions. Radiographs confirmed the absence of fractures, and CT angiography demonstrated preserved arterial flow, excluding major vascular injury.

**Figure 2:**
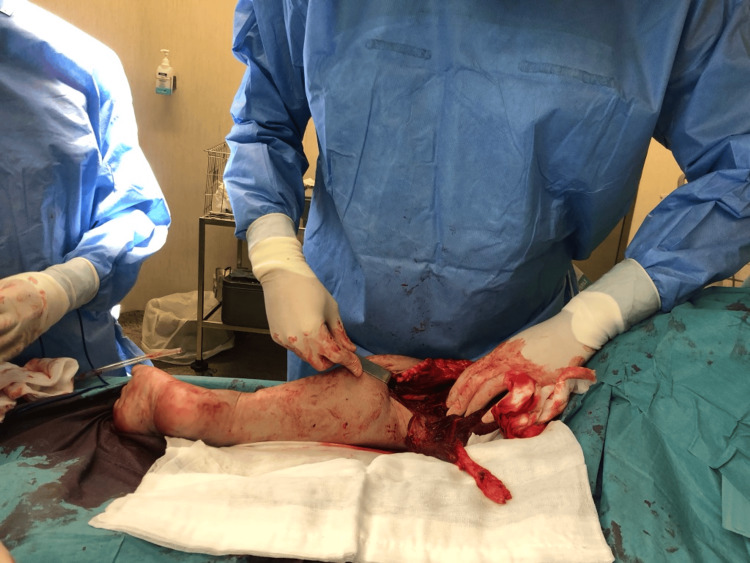
Surgical approach and exploration of the exposed popliteal fossa injury.

**Figure 3:**
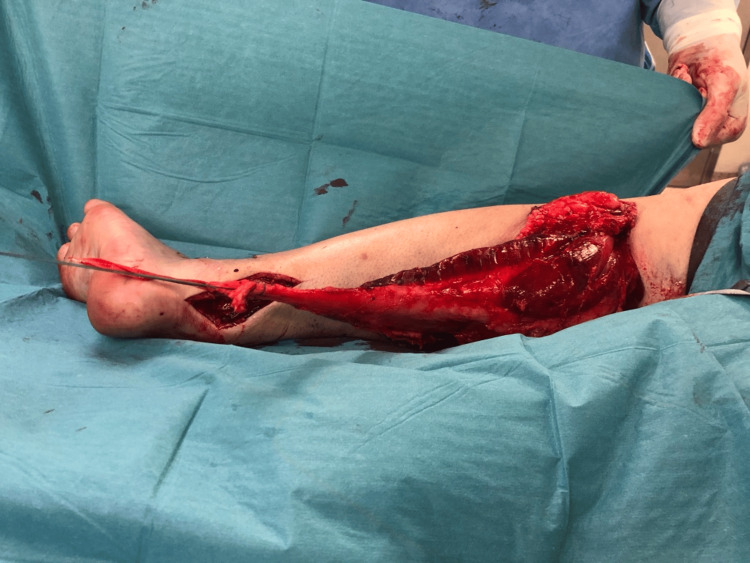
Preparation of the Achilles tendon with Krackow sutures prior to subcutaneous tunnelling along the posterior compartment of the leg.

**Figure 4:**
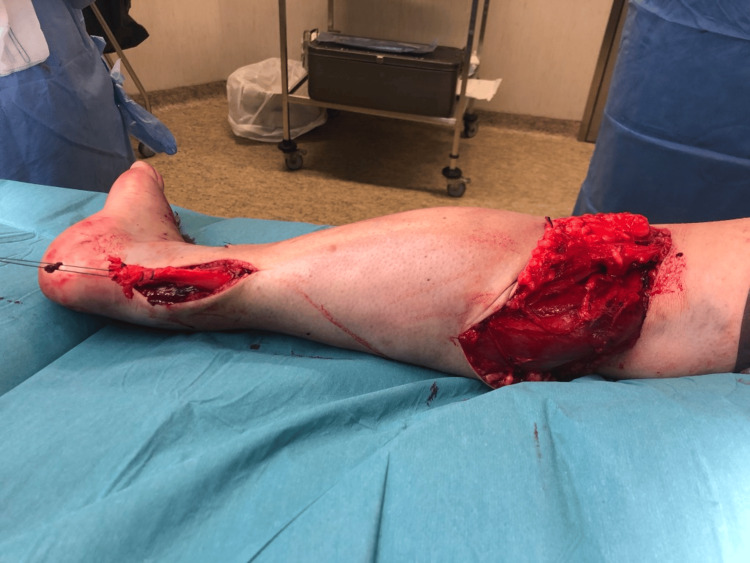
Subcutaneous tunnelling and preparation for reinsertion of the Achilles tendon into the calcaneus.

Surgery was undertaken approximately four hours after the injury. Through direct exposure of the popliteal region (Figure 2), a partial laceration of the popliteal vein was identified and repaired with 6-0 polypropylene. The avulsed tendon and gastrocnemius-soleus complex were isolated and controlled with 0 polyglactin. The tendon had avulsed completely from its calcaneal insertion and extruded proximally through the popliteal wound; because surgery was performed early, no proximal contracture had developed. A subcutaneous tunnel was created along the full length of the posterior compartment, from the popliteal wound to the calcaneal insertion, to re-establish the tendon’s anatomical course (Figures 3, 4). A separate distal approach was made over the calcaneus, where the insertion site was debrided; no distal tendon stump was present, confirming a complete avulsion. The tendon was prepared with Krackow sutures using No. 5 braided polyester suture and secured to the calcaneal tuberosity with two 3.5-mm polyether-ether-ketone suture anchors, and the peritendinous tissue was closed over the repair. The popliteal fossa wound and the distal reinsertion approach were then closed primarily over viable, debrided margins. At the end of the procedure, two prophylactic fasciotomies of the lateral leg compartment and the forefoot were performed to prevent compartment syndrome, given the high-energy crush mechanism and the extensive soft-tissue injury, and were deliberately left open for delayed closure.

Perioperative antibiotic prophylaxis consisted of intravenous cefazolin every eight hours for 24 hours, and the wounds were reviewed at 48 hours, before discharge, and again at two weeks for suture removal. The posterior wound, through which the tendon had extruded, subsequently developed necrosis; this was the indication for delayed skin grafting, which was performed by the plastic surgery team and achieved definitive coverage. The prophylactic fasciotomy wounds were managed by delayed closure, and all wounds were fully healed by the two-year review.

The limb was initially immobilised in 20° of plantarflexion in a posterior splint for two weeks (non-weight-bearing). From weeks 2 to 6, the patient used a walker boot with four heel wedges, starting partial weight-bearing as tolerated and removing one wedge per week, targeting neutral plantarflexion by week 6. The active range of motion focused on plantarflexion, inversion, and eversion within comfort, while dorsiflexion past neutral was avoided until week 6. From weeks 6 to 12, the boot was weaned, and progressive strengthening prescribed by physical medicine and rehabilitation emphasised calf isometrics, concentric raises, eccentric loading, balance work, and gait normalisation. From three to six months, the programme advanced to single-leg heel raises, Alfredson-style eccentric loading, plyometric readiness testing, and return-to-activity training under physiotherapy supervision. This staged plan aligns with contemporary Achilles repair protocols, favouring early protected mobilisation and criterion-based loading [8].

The patient reported being satisfied with the outcome and understood that the residual limitations corresponded to the severity of the injury.

**Figure 5:**
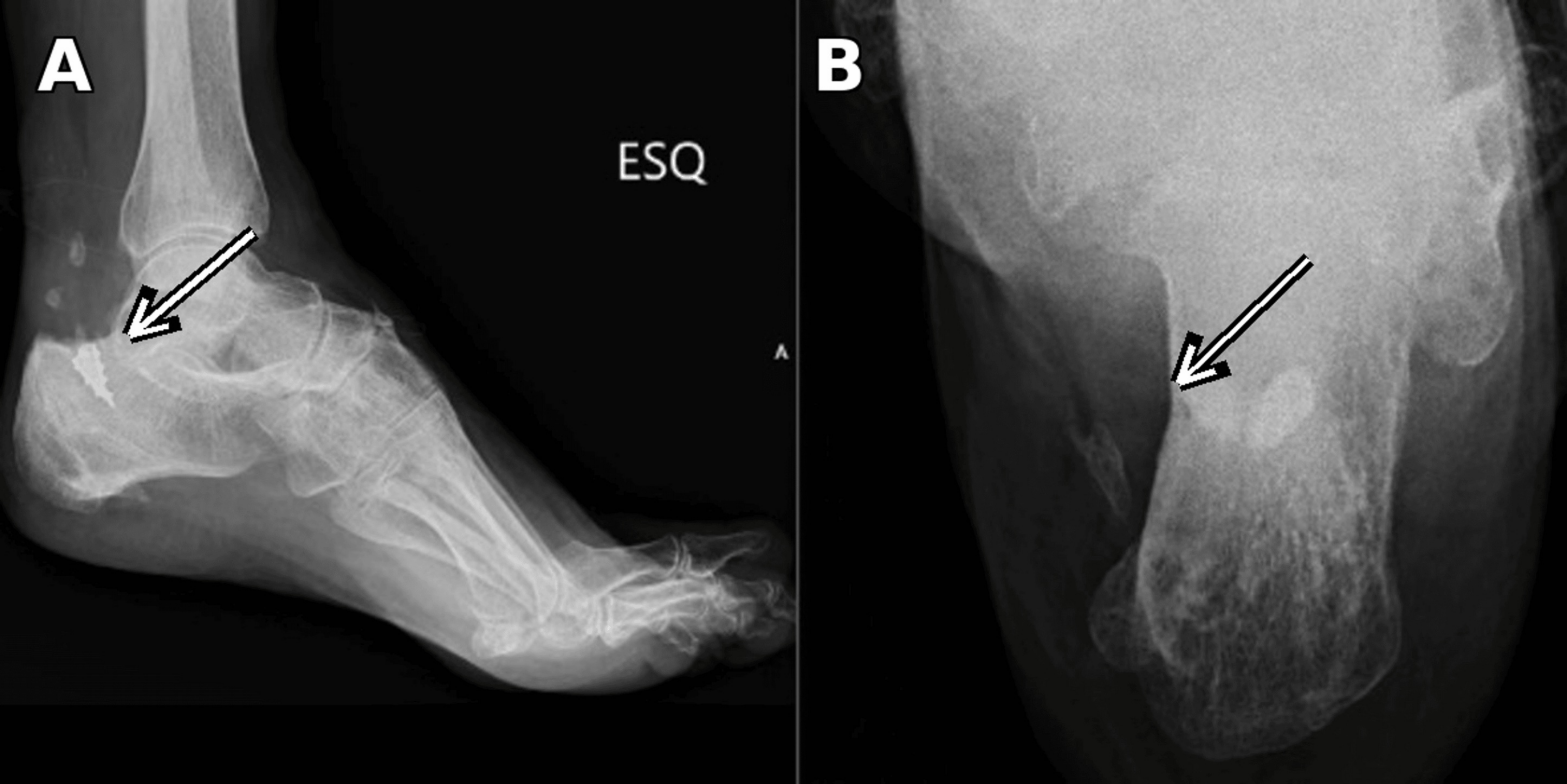
Two-year follow-up radiographs: lateral (A) and axial calcaneal (B) views demonstrating maintained tendon reinsertion with the suture anchors in place (arrows) and no degenerative change or calcaneal deformity.

At two years, functional assessment demonstrated an Achilles Tendon Total Rupture Score (ATRS) of 72/100 and an American Orthopaedic Foot & Ankle Society (AOFAS) Ankle-Hindfoot score of 77/100 (fair). Objective calf endurance testing showed six single-leg heel raises on the injured side compared with 16 on the contralateral side. Control radiographs demonstrated a well-integrated tendon reinsertion with no evidence of bony involvement or late calcaneal deformity (Figure 5).

## Discussion

To date, no reports have described an Achilles tendon avulsion with extrusion through the popliteal fossa in the absence of associated skeletal injury. Most documented Achilles injuries involve distal insertions or mid-substance ruptures, and avulsion from the gastrocnemius-soleus origin has only recently been characterised as a distinct, albeit rare, injury variant [4]. Open Achilles tendon injuries have been described in the context of pure ankle or subtalar dislocations and as part of multistructural trauma patterns [9]. However, these typically involve injuries localised around the ankle or heel. None, to our knowledge, documents tendon retraction and extrusion proximally through the popliteal region. Case series of open Achilles injuries secondary to penetrating trauma or traffic accidents have largely focused on repair techniques, soft-tissue challenges, and the risk of infection [6,7], but do not address the anatomical disruption encountered in our patient.

The proximal trajectory of tendon retraction warrants specific anatomical and biomechanical consideration. In a typical mid-substance rupture, the proximal stump retracts only a limited distance because the gastrocnemius-soleus complex remains tethered within an intact superficial posterior compartment, and the surrounding deep crural fascia and paratenon constrain migration. In our patient, the combination of a high-energy run-over mechanism and a degloving soft-tissue injury disrupted these fascial boundaries, removing the normal constraints to proximal migration. We postulate that a violent dorsiflexion force applied to a loaded triceps surae caused failure at or near the musculotendinous origin rather than at the mid-substance, while the concurrent fascial and compartmental disruption created a low-resistance proximal plane, directly continuous with the popliteal fossa, along which the unopposed elastic recoil of the muscle belly carried the avulsed tendon. The intact bony architecture indicates that the energy was dissipated entirely through soft tissue, an unusual feature given the magnitude of the trauma. Because conventional clinical tests for Achilles rupture (e.g., the Thompson and Matles tests) are unreliable or inapplicable in open injuries, and given the proximity of the wound to the popliteal neurovascular bundle, cross-sectional vascular imaging is essential; we recommend CT angiography in all posterior knee degloving injuries to exclude occult vascular compromise before reconstruction.

From a surgical standpoint, our approach combined urgent vascular control, anatomical tendon reinsertion, and careful soft-tissue management. We favoured reinsertion with suture anchors because tendon quality was adequate after debridement, there was no segmental tendon loss requiring bridging, and early surgery allowed restoration of length and tension while limiting additional donor-site morbidity in a compromised soft-tissue envelope [10]. Where a viable proximal muscle-tendon unit is displaced from its anatomical bed but not segmentally deficient, as in this case, subcutaneous tunnelling along the posterior compartment is a useful technical trigger, re-establishing the tendon’s anatomical course and physiological tension without further soft-tissue stripping [7]. Alternative strategies in more extensive tendon loss include tendon transfers (e.g., flexor hallucis longus), graft augmentation, or staged reconstruction with orthoplastic coverage, none of which were required for tendon reconstruction given the intraoperative findings [11]. The principal soft-tissue challenge arose postoperatively: the posterior wound through which the tendon had extruded developed necrosis and required delayed skin grafting by the plastic surgery team for definitive coverage, underscoring the value of early orthoplastic collaboration in these injuries.

No existing classification fully captures this injury. The Gustilo-Anderson system was designed for open fractures and cannot be directly applied to an isolated soft-tissue injury [12], while the Oestern-Tscherne classification grades the soft-tissue envelope of closed fractures and likewise presupposes an associated bony injury [13]. Our patient therefore falls outside both frameworks: a high-energy, open, degloving soft-tissue injury with complete tendon avulsion but intact bony architecture. Nevertheless, principles drawn from severe open-fracture care-early debridement, multidisciplinary orthoplastic planning, and staged soft-tissue coverage when required-remain applicable [7,12], and this case illustrates how high-energy trauma can produce a soft-tissue wound of severity comparable to an open fracture. Among rarer Achilles variants, sleeve avulsions associated with Haglund’s deformity have been reported [5], but none share the combination of proximal extrusion, complete tendon avulsion, and preserved bony architecture seen here.

Interpreting the functional outcome is complicated by the absence of pre-injury baseline scores and by the scarcity of published outcome data for comparably severe open Achilles injuries, which limits benchmarking against an established natural history. At two years, the patient’s ATRS of 72 approached the population-based Patient Acceptable Symptom State (PASS) threshold of 75 estimated for the ATRS [14], suggesting a symptom state close to that which most patients consider acceptable, despite a measurable strength deficit (six versus sixteen single-leg heel raises). Because the minimal clinically important difference is a change metric, it could not be computed in the absence of a baseline score; the PASS, an absolute threshold, is therefore the more appropriate interpretive anchor here. The persistent plantarflexion endurance deficit is consistent with the magnitude of the initial muscle-tendon injury and characterises a stable chronic-sequelae stage rather than ongoing deterioration. Overall, limb salvage with a near-acceptable patient-reported symptom state and a maintained reinsertion at two years represents a favourable outcome given the unprecedented severity of the injury.

Limitations

This report describes a single patient, and generalisability is therefore limited. Functional outcomes may be influenced by patient factors, rehabilitation adherence, and the extent of associated muscle injury. Pre-injury baseline scores are unavailable, precluding within-patient change analysis and comparison against minimal clinically important difference thresholds, and strength testing relied on pragmatic clinical measures (single-leg heel-raise repetitions) rather than isokinetic dynamometry. These factors should be considered when extrapolating management recommendations.

## Conclusions

This case documents a previously undescribed pattern of Achilles tendon avulsion with proximal extrusion through the popliteal fossa without associated fracture. Beyond its rarity, it offers transferable lessons: posterior knee degloving injuries warrant CT angiography to exclude vascular compromise, and subcutaneous tunnelling provides a reliable means of restoring tendon length and course in a displaced but non-deficient muscle-tendon unit within a compromised soft-tissue envelope. Early multidisciplinary (orthoplastic) management and transparent reporting of operative details and postoperative milestones, together with standardised, objective outcome assessment, ideally including baseline capture and instrumented strength testing where feasible, should be the goal of future reports of such rare high-energy soft-tissue injuries, as underscored by the measurement gaps acknowledged in the present case.
